# Brain Activity of Adolescents with High Functioning Autism in Response to Emotional Words and Facial Emoticons

**DOI:** 10.1371/journal.pone.0091214

**Published:** 2014-03-12

**Authors:** Doug Hyun Han, Hee Jeong Yoo, Bung Nyun Kim, William McMahon, Perry F. Renshaw

**Affiliations:** 1 Department of Psychiatry, Chung Ang University Hospital, Seoul, South Korea; 2 Department of Psychiatry, Seoul National Bundang Hospital, Seoul, South Korea; 3 Department of Psychiatry, Seoul National Hospital, Seoul, South Korea; 4 Department of Psychiatry, University of Utah, Salt Lake City, Utah, United States of America; 5 The Brain Institute, University of Utah, Salt Lake City, Utah, United States of America; Yale University, United States of America

## Abstract

Studies of social dysfunction in patients with autism spectrum disorder (ASD) have generally focused on the perception of emotional words and facial affect. Brain imaging studies have suggested that the fusiform gyrus is associated with both the comprehension of language and face recognition. We hypothesized that patients with ASD would have decreased ability to recognize affect via emotional words and facial emoticons, relative to healthy comparison subjects. In addition, we expected that this decreased ability would be associated with altered activity of the fusiform gyrus in patients with ASD. Ten male adolescents with ASDs and ten age and sex matched healthy comparison subjects were enrolled in this case-control study. The diagnosis of autism was further evaluated with the Autism Diagnostic Observation Schedule. Brain activity was assessed using functional magnetic resonance imaging (fMRI) in response to emotional words and facial emoticon presentation. Sixty emotional words (45 pleasant words +15 unpleasant words) were extracted from a report on Korean emotional terms and their underlying dimensions. Sixty emoticon faces (45 pleasant faces +15 unpleasant faces) were extracted and modified from on-line sites. Relative to healthy comparison subjects, patients with ASD have increased activation of fusiform gyrus in response to emotional aspects of words. In contrast, patients with ASD have decreased activation of fusiform gyrus in response to facial emoticons, relative to healthy comparison subjects. We suggest that patients with ASD are more familiar with word descriptions than facial expression as depictions of emotion.

## Introduction

### Language comprehension and facial affect recognition in patients with autism spectrum disorder

Autism is a developmental disorder characterized by deficits in three domains, including social reciprocity; early language and communication problems; and restrictive, repetitive and stereotyped behaviors [1]. Among these deficits, particular interest in social dysfunction has been focused on the perception of emotional words and facial affect in patients with autism spectrum disorder (ASD) [2]–[6]. Social dysfunction has also been associated with impairment of social perception and cognition, as demonstrated by observation of emotions, actions, body movements, hand gestures, and facial expressions [2]. In addition, social dysfunction has been related to early language and communication impairments in children with ASD [3], [5]. In the domains of comprehension and use of pragmatic language, patients with ASD have been reported to demonstrate impairments in understanding non-literal meaning, such as emotion, humor, and metaphor [7], [8].

Patients with ASD are also known to have impairments in their ability to recognize unfamiliar faces, compared to healthy subjects [9]. Moreover, defects in the perception of facial expression may be a core feature of social deficits in patients with ASD [4]. Patients with autism were impaired in their ability to recognize appropriate facial expressions in videotaped gestures, vocalizations and contexts [10]. Similarly, patients with Asperger's syndrome had difficulty in the comprehension and production of facial and language expressions of emotion [6].

### Facial Emoticons: simplified facial emotion expression

The emoticon in computer-mediated communication may be used for the purpose of facilitating non-verbal emotional communication and for reinforcing the verbal parts of a message [11]. Although facial emoticons differ from facial expression, simplified text or cartoon facial expressions (emoticons) are frequently used in the context of social communication [11], [12]. Lo [12] reported that emoticons, as “quasi-nonverbal cues”, help most people perceive appropriate emotion, attitude, and intention during internet use. Based on these observations, there have also been several empirical results showing that the emoticon is associated with high levels of emotional abstraction [13] and non-verbal information recognition [14], [15]. Interestingly, patients with ASD, relative to healthy comparison subjects, do not apply different strategies for perceiving cartoon faces, while they use different strategies during perception of real human faces [16], [17]. While healthy comparison subjects used a configural strategy in assessing both real and cartoon faces, patients with ASD used a figural strategy in viewing cartoon faces and a local strategy in evaluating real faces [16]. In an assessment of attentional bias using saccade-related, event-related potentials (ERPs), patients with ASD showed a smaller interval in attentional time between faces and objects, relative to healthy comparison subjects [18]. Moreover, patients with ASD can fixate on cartoon characters longer than on real objects [17]. In an fMRI study of emoticon perception, the right inferior frontal gyrus of healthy volunteers, which has been associated with non-verbal communication, was activated in response to viewing facial emoticons [14].

### Fusiform gyrus, language comprehension, and face recognition

Over the last decade, several neuroimaging studies have reported activation in focal brain areas in response to semantic comprehension of language and human facial affect recognition in healthy subjects. Bookheimer [19] reported that inferior frontal and temporal cortex play a crucial role in the comprehension of language in healthy subjects. Ghosh et al. [20] noted that contextual integration in lexical processing was associated with activation of the left fusiform gyrus. In a review of studies of the role of the fusiform gyrus in face recognition, Kanwisher and Yovel [21] suggested that the fusiform gyrus is specific for face recognition in terms of detecting and extracting the necessary perceptual information to recognize the face. Similarly, the fusiform gyrus is well known to be dysfunctional in prosopagnosia patients [22]. In a review of fMRI articles, Sabatinelli et al. [23] suggested that the early developmental failure of the ventral temporal area (amygdala-fusiform system) was associated with deficits in social perception and social cognition. However, in fMRI studies, there have been reports that the fusiform gyrus was not activated in response to viewing facial expressions of emotion in patients with ASD [24], [25]. Also, while a healthy control group showed activation of right fusiform gyrus in response to a discrimination of facial expression task, patients with autism did not show activation [26].

### Hypothesis

We hypothesized that patients with ASD would have decreased ability to recognize affect via emotional words and facial emoticons, relative to healthy comparison subjects. In addition, we expected that this decreased ability would be represented by altered activity of the fusiform gyrus in patients with ASD.

## Method

### Subjects

In response advertisements posted in Chung Ang University Hospital and other local hospitals, fifteen adolescents with autism spectrum disorders were screened. Inclusion criteria included: adolescents between the ages of 13 years to 18 years; diagnosed as having autism spectrum disorder; IQ≥70, ADOS score = 4–7 [27]. Exclusion criteria included: Comorbid Axis I Disorders, as determined by Korean Kiddie Schedule for Affective Disorders and Schizophrenia-Present and Life time version (K-SADS-PL) [28]; history of head trauma with loss of consciousness, seizure disorder, multiple sclerosis, brain tumor, claustrophobia, metal implantation or cerebrovascular accident; serious or chronic medical illness; IQ<70; and a history of substance abuse. Of these fifteen adolescents, three adolescents had IQ<70 and two adolescents were excluded due to co-morbid major depressive disorder and/or attention deficit hyperactivity disorder. Finally, 10 adolescents with ASD and 10 age and sex matched healthy comparison subjects have been recruited. The research protocol was approved by the Chung Ang University Hospital Institutional Review Board. Written informed assent was provided by adolescents and written informed consent was provided by parents.

### Assessment

The diagnosis of autism was further evaluated with the ADOS [29] by social worker B.G.Y. who has both clinician and research certificates for ADOS and Autism Diagnostic Interview (ADI) based on training by supervisor Catherine Lord. The clinical symptoms were assessed by the child's mother using the Childhood Autism Rating Scale (CARS) parent version. IQ scores were obtained for all participants using the Korean Wechsler Adult Intelligence Scale (K-WAIS) [30]. Social communication was evaluated by the mother using the Social Communication Questionnaire (SCQ)-current form [31]. The inter-item consistency of SCQ Korean version has been reported to range from 0.84 to 0.93 [32].

Brain activity in both ASD patients and healthy subjects was assessed, in two separate scan sequences, in response to (1) emotional words and (2) scenes depicting emoticons in the scanner. All MR imaging was performed using 3 T blood oxygen level dependent (BOLD) functional magnetic resonance imaging (fMRI, Achieva 3.0, Philips, Eindhoven, the Netherlands). The stimulation was presented through an IFIS-SA™ system (MRI Device Corporation, Waukesha, WI, USA) during a single fMRI scanning session. For the fMRI session, 180 echo planar images (EPI, 33 transverse slices, 4.0 mm thickness, voxel size of 1.8×1.8×4.0 mm, TE = 30 msec, TR = 3000 ms, Flip angle = 90°, in-plane resolution = 128×128 pixels, field of view (FOV) = 230×230 mm) were recorded at 3-second intervals. For anatomical imaging, 3D T1-weighted magnetization-prepared rapid gradient echo (MPRAGE) data were gathered with these parameters: TR = 2000 ms, TE = 4.00 ms, FOV = 256×256 mm, 340 slices, 0.9×0.9×1.0 mm voxel size, flip angle = 30°.

### Tasks

#### Stimulation 1: Emotional words

**Figure 1:**
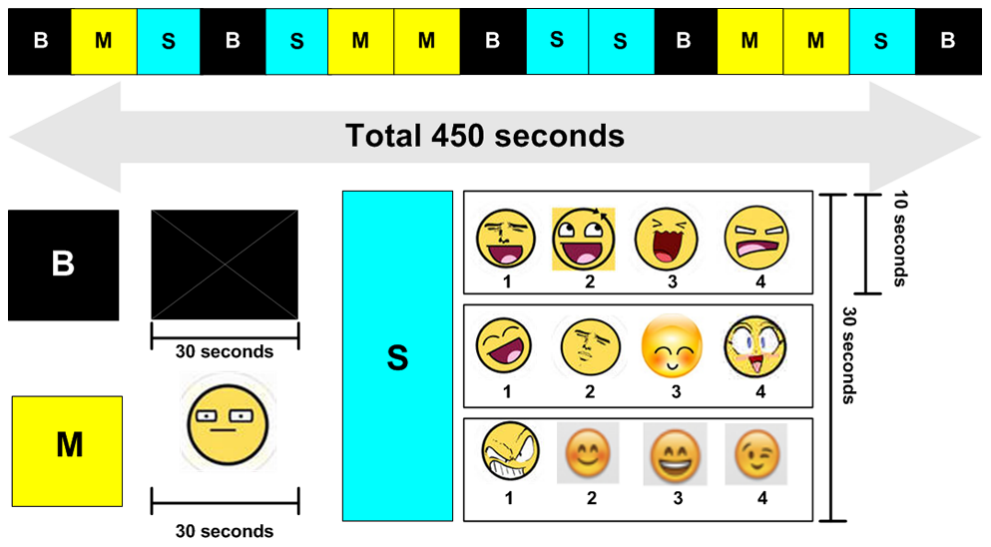
Facial emoticons.

Sixty emotional words (45 pleasant words +15 unpleasant words) were extracted from a report on Korean emotional terms and their underlying dimensions [33]. In ratings by forty young college students with a seven point analogue scale, the prototypicality and familiarity of these sixty words were 4.01–5.77 and 4.16–5.82, respectively [33]. Pleasant score of pleasant words was over 3.0 and the unpleasant score of unpleasant words was over 3.0. Both the pleasant score and unpleasant score in response to non-emotional words were less than 1.0 [33]. While in the scanner, each subject in patient group and healthy control group was asked to identify via microphone the one unpleasant emotional word presented among three pleasant words. Test-retest reliability for identification of emotional words was 0.82. The percentage of correct answers for emotional words in seventy healthy subjects was 81.2±10.3% (minimum = 56.6%, maximum = 96.7%). A video was constructed that was 450-seconds long and consisted of five continuous 90-second segments. Each 90-second segment consisted of three 30-second sub-segments. A white cross on a black background (B), a single non-emotional word (Match, M), and an emotional word stimulation (Stimulation, S) were included in these 90-second segments. The five segments were ordered as follows: B-M-S, B-S-M, M-B-S, S-B-M, and M-S-B. During the M sub-segment, three non-emotional words were presented every ten seconds. During the S sub-segment, three sets of four mixed emotional words were presented every ten seconds (Figure 1).

#### Stimulation2: Emoticon faces

Sixty emoticon faces (45 pleasant faces +15 unpleasant faces) were extracted and modified from on-line sites (http://image.search.naver.com/search.naver, http://forums.themavesite.com/index.php?topic=6103.0)(Figure S3 in File S1). While in the scanner, each subject in patient group and healthy control group was asked to identify via microphone the number of unpleasant face emoticon among three other pleasant faced emotions. Test-retest reliability for facial emoticon identification is 0.84. The percentage of correct answers for facial emoticon identification in seventy healthy subjects was 86.6±9.2% (minimum = 66.7%, maximum = 100%). The sixty emotional words consisted of forty-five pleasant emotional words and fifteen unpleasant emotional words. The video used was 450-seconds long and consisted of five continuous 90-second segments. Each 90-second segment consisted of three 30-second sub-segments. A white cross on a black background (B), a single neutral face (Match, M), and an emoticon stimulation (Stimulation, S) were included in these 90-second segments. The five segments were ordered as follows: B-M-S, B-S-M, M-B-S, S-B-M, and M-S-B. During the M sub-segment, three neutral faces were presented every ten seconds. During the S sub-segment, three sets of four mixed emoticon faces were presented every ten seconds (Figure 1).

### fMRI data analysis

Acquired fMRI data was analyzed by the Brain Voyager software package (BVQX 1.9, Brain Innovation, Maastricht, The Netherlands). In a multi-scale algorithm, each fMRI time series was registered to the MPRAGE 3D data set. The anatomic images were spatially normalized to standard Talairach space [34]. The time series data was also spatially normalized. Scan time correction and motion correction were applied during processing. Spatial smoothing using 6 mm FWHM Gaussian kernel and temporal smoothing using a 4 second Gaussian kernel were also applied. Head motion algorithms represent head movements by 6 parameters, three translation (displacement) parameters and three rotation parameters. Six parameters are estimated iteratively by analyzing how a source volume should be translated and rotated in order to better align with the reference volume. Head motion of less than 3.5 mm (translation) or 3.5° (rotation) relative to the target volume was considered to be acceptable. None of the ASD and healthy subjects was excluded due to excessive head movement in the present study. There was no difference in head motion between ASD patients and healthy comparison subjects (Table S1 and Figure S1 in File S1).

### Statistical analysis

Differences in demographic data including age, IQ, CARS scores, SCQ scores, and percentage of correct answers in identifying emotional words/emoticons between ASD and healthy subjects were compared using the Mann-Whitney U test and ANCOVA test controlling for IQ. For the analysis of fMRI signal time-courses, the general linear model (GLM) and random effects analysis (RFX) were applied to construct individual and group statistical parametric maps of brain activation. Activations were computed by contrasting the S sub-segments (emotional words/emoticons) to their corresponding M sub-segments (neutral words/neutral emoticons). With that parametric maps, ANCOVA tests controlling for IQ were applied to identify clusters at a level of FDR<0.05, voxels >40. The Talairach code of clusters was identified by nearest coordinate in Talairach daemon [34].

As a second-level analysis in subjects with ASD, the correlations between mean β value in clusters and the mean SCQ scores were analyzed using partial correlations controlling for IQ, ADOS-communication score, and ADOS-social score. In order to account for multiple comparisons, we set significant p values  = 0.0166 (0.05/3, CARS, SCQ, and correct response rates).

## Results

### Demographic characteristics

There were significant differences between ASD subjects and healthy comparison subjects in the IQ (U = 3.5, z = 3.5, p<0.01), CARS score (F = 15.5, df = 1, p<0.01), SCQ score (F = 53.4, df = 1, p<0.01), and the percentage of correct answers in identifying emotional words (F = 32.7, df = 1, p<0.01) and emoticon faces (F = 10.1, df = 1, p<0.01)(Table 1)(Figure S2 in File S1). The mean IQ values of the healthy subjects and the ASD subjects were 105.5±8.9 and 81.5±9.0, respectively. The correct response rates for emotional words in healthy subjects and ASD subjects were 86.4±8.4 and 40.3±11.6%, respectively. The correct response rates for facial emoticons in the healthy subjects and ASD subjects were 90.3±9.1 and 53.0±14.6%, respectively.

**Table 1:** Demographic Characteristics.

Subjects	Age (Mo)	Sex	ADOS			CARS	SCQ	IQ		
			A	B	Diagnosis			FSIQ	VIQ	PIQ
Patients with ASD										
ASD 1	204	F	3	4	ASD	21	22	88	92	74
ASD 2	198	M	2	9	ASD	26.5	29	77	82	83
ASD 3	202	M	3	9	Autism	23	26	71	75	74
ASD 4	215	F	5	13	Autism	37.5	14	80	77	82
ASD 5	205	M	4	12	Autism	29	16	71	76	62
ASD 6	158	M	4	11	Autism	30.5	18	79	73	84
ASD 7	160	M	5	6	Autism	34.5	22	85	90	75
ASD 8	209	M	3	6	ASD	21	23	75	73	74
ASD 9	212	M	4	9	Autism	26.5	25	93	96	89
ASD 10	161	M	4	4	ASD	21.5	22	78	72	81
mean±SD^a^	192.4±23.1	8M/2F				27.1±5.8	21.7±4.6	81.5±8.9	80.6±8.9	77.8±7.6
Healthy Control Subjects										
HC1	191	M	-	-	Healthy	16	3	100	103	95
HC2	202	M	-	-	Healthy	15	2	110	110	107
HC3	216	M	-	-	Healthy	16.5	4	117	115	116
HC4	156	F	-	-	Healthy	15.5	3	116	125	99
HC5	155	M	-	-	Healthy	15	2	109	114	100
HC6	118	M	-	-	Healthy	15	1	109	108	109
HC7	158	M	-	-	Healthy	17	2	94	97	92
HC8	214	M	-	-	Healthy	15	1	94	90	102
HC9	178	M	-	-	Healthy	15.5	2	93	97	89
HC10	190	F	-	-	Healthy	15	3	120	119	116
mean±SD^b^	178.8±32.5	8M/2F				15.5±0.7	2.3±0.9	106.2±10.2	107.8±8.9	102.5±9.4
Statistics	U = 33.0, z = 1.3, p = 0.19					F = 15.5, df = 1, p<0.01	F = 53.4, df = 1, p<0.01	U = 3.50, z = 3.51, p<0.01	U = 2.50, z = 3.59, p<0.01	U = 03.50, z = 3.74, p<0.01
a vs b										

Mann-Whitney U test, SCQ: Social Communication Questionnaire, FSIQ: IQ total, VIQ: verbal IQ, PIQ: performance IQ, A: ADOS-A (Autism Diagnostic Observation Schedule-A): communication score, B: ADOS-B: social score, the threshold scores for a diagnosis of autism on the ADOS are 3 and 6 for communication and social symptoms, respectively. Threshold scores for a less severe diagnosis of ASD on the ADOS are 2 and 4 for communication and social symptoms, respectively. Mo: months, CARS: Child Autism Rating Scale, ASD: autism spectrum disorders, HC: healthy comparison subjects.

Correct responses to emoticon were positively correlated with the correct response to emotional words (r = 0.91, p<0.01). There were no significant correlations between IQ and correct response of emotional words or emoticon. Controlling for IQ, the mean SCQ score were negatively correlated with the correct response to emotional words (r = −0.77, p = 0.01) in ASD patients. Controlling for IQ, SCQ score was negatively correlated with the correct responses to emoticons at a trend level (r = −0.73, p = 0.02).

### Clusters in the interaction between emotional words and subject factors at baseline

**Figure 2:**
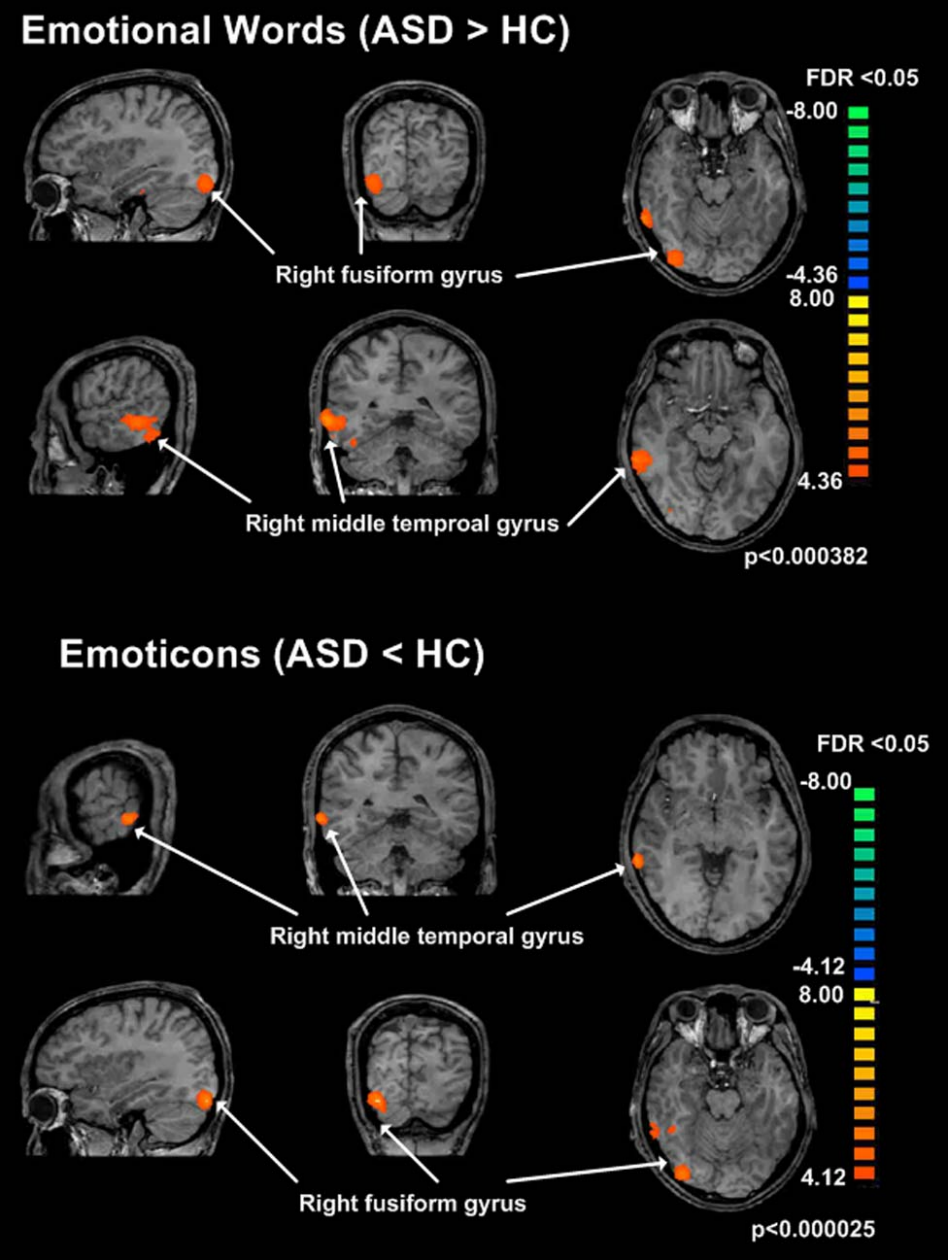
Brain activity in response to emotional words / facial emoticons. Brain activity in response to emotional words (ASD > healthy subjects), Cluster1 (CL1): right temporal lobe, fusiform gyrus, Brodmann area 20; Cluster2 (CL2): Right Cerebrum,Occipital Lobe,Fusiform Gyrus, Right Cerebrum, Occipital Lobe, Inferior Occipital Gyrus, Right Cerebellum, Posterior Lobe. Brain activity in response to emoticons (ASD < healthy subjects), Cluster3 (CL3): Right Cerebrum,Temporal Lobe, Middle Temporal Gyrus, Cluster4 (CL4): Right Cerebellum, Posterior Lobe, Right Cerebrum,Occipital Lobe, Fusiform Gyrus.

**Figure 3:**
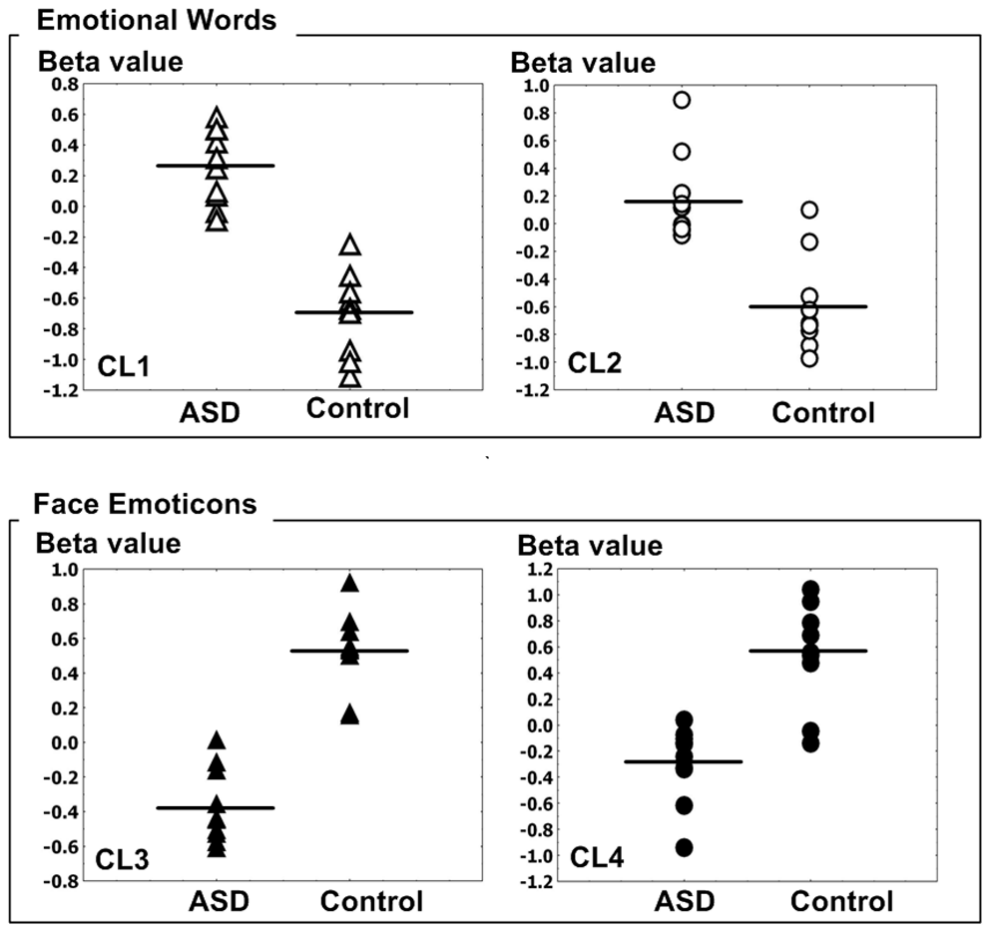
The beta values of brain activity in response to emotional words / facial emoticons. Cluster1 (CL1, unfilled triangle): right temporal lobe, fusiform gyrus, Brodmann area 20; Cluster2 (CL2, unfilled circle): Right Cerebrum,Occipital Lobe,Fusiform Gyrus, Right Cerebrum, Occipital Lobe, Inferior Occipital Gyrus, Right Cerebellum, Posterior Lobe, Cluster3 (CL3, filled triangle): Right Cerebrum,Temporal Lobe, Middle Temporal Gyrus, Cluster4 (CL4, filled circle): Right Cerebellum, Posterior Lobe, Right Cerebrum,Occipital Lobe, Fusiform Gyrus.

In an F test, interaction between group (ASD vs. Healthy subjects) and stimuli (Emotional words vs. Neutral words), two clusters of activity in patients with ASD were increased, compared to healthy subjects (FDR<0.05, p<0.00038); Cluster1 (CL1): Talairach x, y, z; 55,−39, −9, voxels  = 242, right temporal lobe, fusiform gyrus, Brodmann area 20; Cluster2 (CL2): 32, −80, −17, voxels  = 70, Right Occipital fusiform Gyrus, Brodmann area 19 (Figure 2)(Figure 3)(Table2). In an F test, interaction between group (ASD vs. Healthy subjects) and stimuli (Emotional words vs. Neutral words), there were no significant clusters of activity in patients with ASD that were decreased, compared to healthy subjects (FDR<0.05, p<0.00038).

**Table 2:** Clusters in the interaction between emotional words, emoticons and subject factors at baseline.

Talairach Code			voxels	t	p	regions
x	y	z				
Clusters in the interaction between emotional words and subject factors (ASD>healthy subjects)						
32	−80	−17	70	4.36	FDR <0.05, P<0.000382	Right Fusiform Gyrus, BA 19
55	−39	−9	242	4.36	FDR <0.05, P<0.000382	Right Middle Temporal Gyrus, BA 20
Clusters in the interaction between facial emoticons and subject factors (ASD<healthy subjects)						
61	−39	−4	40	5.62	FDR <0.05, P<0.000025	Right Middle Temporal Gyrus, BA 21
31	−82	−20	65	4.12	FDR <0.05, P<0.000643	Right fusiform Gyrus, BA 18

ASD: patients with autism spectrum disorder, FDR: false discovery rate, BA: Brodmann Area.

### Clusters in the interaction between facial emoticons and subject factors at baseline

In an F test, interaction between group (ASD vs. Healthy subjects) and stimulus (Emoticons vs. Neutral faces), two clusters of activity in patients with ASD were decreased (FDR<0.05, p<0.000025); Cluster3 (CL3): Talairach x, y, z; 61, −39, −4, Right Cerebrum, Temporal Lobe, Middle Temporal Gyrus, Brodmann area 21, Cluster4 (CL4): Talairach x, y, z; 31, −82, −20, Right Cerebellum, Posterior Lobe, Right Occipital fusiform Gyrus, Brodmann area 18 (Figure 2)(Figure 3)(Table2). In an F test, interaction between group (ASD vs. Healthy subjects) and stimulus (Emoticon vs Neutral faces), there were no significant clusters of activity in patients with ASD that were increased, compared to healthy subjects (FDR<0.05, p<0.000025).

### Correlations between Social Communication Questionnaire (SCQ) and brain activity

**Figure 4:**
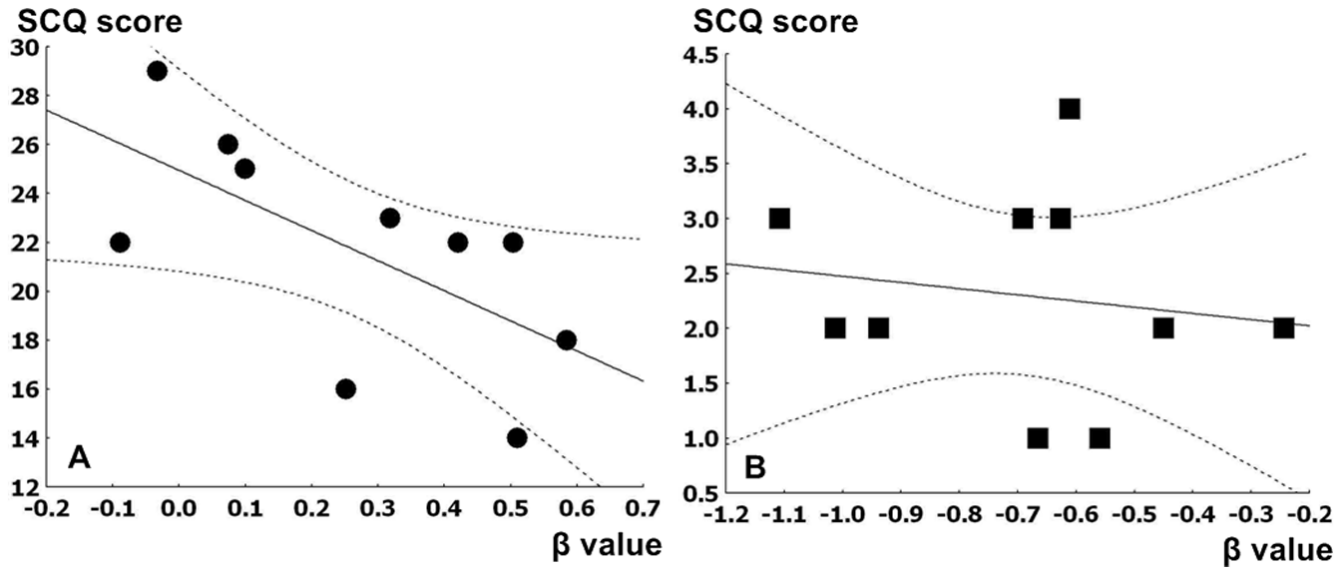
Correlation between mean SCQ score and the mean beta value within right temporal fusiform gyrus. A: The correlation between mean SCQ score in ASD patients and the mean beta value within right temporal fusiform gyrus in response to emotional words, with a partial correlation controlling for IQ, r = −0.82, p = 0.015. B: The correlation between mean SCQ score in healthy control subjects and the mean beta value within right temporal fusiform gyrus in response to emotional words, with a partial correlation controlling for IQ, r = −0.16, p = 0.67.

The mean SCQ score in ASD patients was negatively correlated with the mean beta value of right temporal fusiform gyrus in response to emotional words (r = −0.82, p = 0.02)(Figure 4). There were no significant correlations between SCQ scores, CARS scores, correct responses on emotional words/emoticon tests and the beta value of other clusters in response to emotional words. Similarly there were no significant correlations between SCQ scores, CARS scores, correct responses on emotional words/emoticon tests, and the beta value of other clusters in response to facial emoticons. There was no significant correlation between CARS score and other clusters.

## Discussion

As expected, patients with ASD showed lower correct response rates for recognizing affect via emotional words and facial emoticons. However, there were divergent patterns of brain activation for emotional words and facial emoticons. The fusiform gyrus in the patients with ASD activated to a greater extent during emotional word tasks, relative to the healthy comparison subjects. Conversely, the activity of fusiform gyrus of patients with ASD was lower in response to facial emoticons, compared to healthy subjects.

Difficulty in recognizing emotional and non-verbal meanings in words and facial affect in patients with ASD has been reported in several studies [2]–[6]. Moreover, the decreased recognition of emotion and non-verbal meanings has been associated with increased activity of fusiform gyrus in patients with ASD [20], [24], [25]. In solving verbal and visual problems, the dissociation between verbal and visuo-spatial abilities in terms of reduced activation of fronto-temporal language areas and increased activation of occipito-parietal and ventral temporal circuits has been noted in patients with ASD [35], [36]. In solving verbal problems, patients with ASD may use the right hemisphere as compensation for a dysfunctional left hemisphere [37]–[39]. In a study of speaker-incongruent sentences vs. speaker-congruent sentences, patients with ASD showed increased activation in right inferior frontal gyrus, compared to healthy controls [38]. During pragmatic language comprehension, increased activation in the right inferior frontal gyrus in patients with ASD has also been observed [37], [39]. Taken together, we hypothesize that the fusiform gyrus in the patients with ASD may require greater activation in order to interpret to the meaning of emotional words, compared to healthy subjects. The increased activation within fusiform gyrus in response to emotional words in patients with ASD may be associated with a compensatory mechanism to control social information processing. Dichter et al. [40] have reported that patients with ASD showed greater activation within the anterior cingulate in response to the stimuli of social target detection, compared to healthy subjects. In addition, mean SCQ scores (higher SCQ scores are associated with greater deficits in social communication) were negatively correlated with brain activity within the fusiform gyrus in response to emotional words. However, mean SCQ scores had no correlation with brain activity within any other cluster in response to facial emotion. The observations suggest that patients with ASD may be more familiar with verbal expression than facial expression during emotional communication. Taken together, we hypothesize that the fusiform gyrus in the patients with ASD may require greater activation in order to interpret to the meaning of emotional words, compared to healthy subjects. In other words, that pattern was though as cortical inefficiency during social information processing [41].

As an explanation for the social deficits in patients with ASD, the disability in facial emoticon recognition is thought to be associated with emotional salience [9], [26] or lack of social insight [42]. Simplified carton facial expressions (emoticons) are frequently used in the context of social communication, because emoticons can be used as a surrogate for nonverbal emotional expression [11], [12]. In a study of the role of emoticons in computer-mediated communication, Derks et al. [11] suggested that emoticons are commonly used in a manner similar to facial behavior in face to face communication with respect to social context and interpersonal interaction. In the case of real facial experiments, Schultz et al. [26] reported that patients with ASD were hard put to differentiate even neutral face recognition. Among various social brain areas, the lateral fusiform gyrus or fusiform face area has been thought to be important for the rapid recognition of faces [43]. In face discrimination tasks, patients with ASD showed less activation of the fusiform gyrus, compared to healthy subjects [26]. Patients with ASD are thought to recognize faces in a different manner compared to healthy subjects. They focus more on feature-based than configural analyses [44] and the fusiform gyrus is known to be associated with configural processing [43]. The decreased activation within fusiform gyrus in response to emoticons in patients with ASD may be due to the use of different cognitive strategies to solve problems, relative to healthy subjects. Those differences may be caused by differences in programmed cell death, lack of functional specialization or deviant myelination during synaptogenesis and brain development [45], [46]. Patients with ASD have difficulty with face discrimination, due not to the emotional valence, but to different patterns of facial recognition, compared to healthy subjects. We expect that ASD patients would show a similar neural response following the presentation of one pleasant emoticon out of three unpleasant ones, compared to current paradigm (one unpleasant one out of three pleasant ones).

In response to the emoticon task, right middle temporal gyrus was less activated in ASD patients, compared to healthy subjects. This brain region has been implicated in the pathophysiology of ASD in several prior studies. Freitag et al. [47] have suggested that the interpretation of complex motion may be associated with gray matter volumes within the right medial temporal cortex. Alaerts et al. [48] reported that hypoactivity of posterior superior temporal sulcus might be linked with the social deficits characteristic of ASD patients. Herrington et al [49] reported that the detection of a point-light walking figure was associated with the activity within inferior frontal gyrus, fusiform gyrus, and amygdala. In an fMRI study with stimulation in response to a biological motion task, Koldewyn et al. [50] reported that ASD patients showed reduced posterior superior temporal sulcus, parietal, and frontal activity, relative to healthy comparison subjects. Taken together, these results may be associated with decreased bodily motion or gesture perception. ASD patients are thought to have decreased visual sensitivity to human movements and gesture comprehension [51].

There are several limitations in the current study. First, the number of participants was too small to allow generalization of the results. Second, spoken responses during scanning may have increased subject motion and altered apparent brain activity [52]. To address this issue, we monitored and corrected for possible motion artifacts. In addition, patients with ASD and healthy control subjects were asked to respond equally in the task (emotional word/emoticon) stimulation. However, the two subject groups performed this emoticon labeling the task with different degrees of accuracy, which is a limitation in terms of comparing the extent of BOLD activation across groups. Finally, we did not measure response times in this experiment.

## Conclusions

The brain response to emotional words in patients with ASD, although requiring a greater degree of fusiform gyrus activation relative to healthy comparisons subjects, is similar to healthy comparison in the manner of activation. In contrast, the fusiform gyrus response to emoticon faces is different from healthy comparison subjects. Patients with ASD have great activation of the fusiform gyrus in response to emotional aspects of words, when compared to healthy subjects. However, relative to healthy comparison subjects, patients with ASD have lower activation of fusiform gyrus in response to emotional meaning in facial emoticons.

## Supporting Information

File S1 **This file contains Table S1 and Figure S1-Figure S3.** Table S1,Translation and Rotation during scans. Figure S1, Head motion correction. Figure S2, Comparison of behavioral scores between ASD patients and healthy comparison subjects. Figure S3, Face emoticons.
(DOCX)
