# Supplementary material for: Brain Activity of Adolescents with High Functioning Autism in Response to Emotional Words and Facial Emoticons
Source: PLoS One. 2014 Mar 12;9(3):e91214. doi: 10.1371/journal.pone.0091214 (PMC3951306; doi:10.1371/journal.pone.0091214)
Supplement: File S1 — This file contains Table S1 and Figure S1-Figure S3. Table S1,Translation and Rotation during scans. Figure S1, Head motion correction. Figure S2, Comparison of behavioral scores between ASD patients and healthy comparison subjects. Figure S3, Face emoticons. (DOCX) [file pone.0091214.s001.docx]

Table S1. Translation and Rotation during scans

|  | Translation X | Translation Y | Translation Z | Rotation X | Rotation Y | Rotation Z |
| --- | --- | --- | --- | --- | --- | --- |
| Sub1 mean±S.D.  Min/Max | 0.609±0.544  -1.178/1.471 | -0.076±0.152  -0.423/0.281 | -0.611±0.547  -1.380/0.905 | 0.141±0.712  -1.623/1.669 | 0.047±0.115  -0.232/0.326 | -0.015±0.132  -0.294/0.428 |
| Sub2 mean±S.D.  Min/Max | 0.562±0.423  -0.035/1.471 | -0.104±0.069  -0.224/0.094 | -0.552±0.343  -1.223/-0.037 | -0.104±0.133  -0.349/0.409 | 0.009±0.026  -0.069/0.089 | -0.008±0.020  -0.055/0.057 |
| Sub3 mean±S.D.  Min/Max | 0.371±0.221  -0.032/1.131 | -0.112±0.025  -0.221/0.026 | -0.651±0.441  -1.003/-0.067 | -0.115±0.131  -0.259/0.519 | 0.012±0.015  -0.071/0.101 | -0.012±0.018  -0.061/0.0421 |
| Sub4 mean±S.D.  Min/Max | 0.401±0.356  -0.527/1.462 | -0.207±0.092  -0.465/0.095 | -0.657±0.355  -1.174/2.336 | -0.706±0.320  -1.439/1.191 | 0.009±0.122  -0.288/0.223 | 0.031±0.068  -0.427/0.127 |
| Sub5 mean±S.D.  Min/Max | 1.249±0.820  0.041/2.620 | -0.137±0.052  0.268/0.094 | 0.169±0.280  -1.134/0.663 | 0.265±0.206  -0.056/0.578 | 0.091±0.218  -0.196/0.498 | -0.586±0.490  -1.262/0.050 |
| Sub6 mean±S.D.  Min/Max | -0.029±0.227  -0.287/1.471 | 0.072±0.132  -0.498/0.265 | 0.178±0.219  -1.134/0.504 | 1.015±0.537  -0.694/1.901 | 0.048±0.119  -0.318/0.297 | -0.022±0.064  -0.218/0.142 |
| Sub7 mean±S.D.  Min/Max | -0.156±0.231  -0.384/1.471 | 0.302±0.134  -0.001/0.511 | 0.074±0.183  -1.133/0.436 | 0.903±0.433  -0.011/1.624 | 0.384±0.235  -0.100/0.727 | -0.049±0.061  -0.099/0.199 |
| Sub8 mean±S.D.  Min/Max | 1.208±0.722  0.021/2.384 | 0.374±0.146  -0.014/0.571 | -1.019±0.592  -1.960/0.033 | 0.454±0.242  0.076/0.861 | -1.044±0.615  -1.932/-0.049 | -0.540±0.229  -0.830/0.050 |
| Sub9 mean±S.D.  Min/Max | 0.383±0.282  -0.075/0.919 | -0.068±0.076  -0.344/0.049 | -0.288±0.288  -1.023/0.173 | 0.042±0.158  -0.621/0.224 | 0.099±0.102  -0.169/0.299 | -0.082±0.106  -0.266/0.131 |
| Sub10 mean±S.D.  Min/Max | 1.296±0.604  0.052/2.286 | 0.606±0.221  0.004/0.848 | 1.096±0.449  0.022/1.542 | 1.596±0.621  -0.035/2.298 | -0.528±0.194  -0.814/0.072 | -0.838±0.273  -1.146/0.032 |
| **Total mean±S.D.** | **0.589±0.514** | **0.062±0.272** | **-0.226±0.615** | **0.349±0.666** | **-0.087±0.403** | **-0.212±0.315** |
| HC1 mean±S.D.  Min/Max | 0.863±0.633  -0.049/2.285 | -0.008±0.086  -0.095/0.836 | -0.046±0.144  -0.212/1.405 | -0.046±0.145  -0.212/1.405 | -0.041±0.183  -0.159/2.107 | 0.119±0.129  -1.133/0.269 |
| HC2 mean±S.D.  Min/Max | 1.562±0.723  0.035/1.471 | -0.305±0.129  -0.234/0.066 | 0.231±0.313  1.241/0.035 | -0.115±0.133  -0.401/0.509 | 0.109±0.057  -0.123/0.191 | -0.108±0.050  -0.155/0.055 |
| HC3 mean±S.D.  Min/Max | 1.116±0.812  0.031/1.881 | 0.671±0.042  -0.011/0.774 | 1.021±0.592  0.960/0.033 | 0.454±0.242  0.076/0.861 | 0.013±0.249  1.632/0.049 | -0.712±0.131  -0.921/0.151 |
| HC4 mean±S.D.  Min/Max | 1.045±0.656  -0.008/2.259 | -0.149±0.083  -0.384/0.038 | 0.138±0.216  -0.004/0.885 | 0.954±0.516  -0.404/1.847 | 0.154±0.234  -0.713/0.464 | -0.307±0.196  -0.803/0.018 |
| HC5 mean±S.D.  Min/Max | 0.107±0.096  -0.056/0.322 | -0.088±0.048  -0.047/0.191 | -0.687±0.304  0.010/0.954 | 1.149±0.954  -0.713/1.464 | 0.171±0.156  -0.404/0.847 | -0.229±0.277  -0.760/0.071 |
| HC6 mean±S.D.  Min/Max | 0.236±0.312  -0.240/0.816 | 0.048±0.109  -0.149/1.130 | -0.631±0.240  -0.740/0.001 | 0.179±0.251  -0.240/0.559 | 0.437±0.197  -0.029/1.073 | -0.349±0.42  -0.024/0.517 |
| HC7 mean±S.D.  Min/Max | 0.364±0.521  -0.043/1.333 | -0.201±0.039  -0.124/0.081 | -0.451±0.311  -1.003/-0.031 | -0.109±0.233  -0.449/0.309 | 0.011±0.021  -0.054/0.069 | -0.012±0.021  -0.045/0.053 |
| HC8 mean±S.D.  Min/Max | 0.511±0.454  -0.423/1.151 | -0.101±0.112  -0.265/0.113 | -0.551±0.353  -1.001/1.923 | -0.612±0.331  -1.331/1.201 | 0.014±0.111  -0.291/0.423 | 0.043±0.078  -0.322/0.325 |
| HC9 mean±S.D.  Min/Max | -0.236±0.334  -0.4424/1.073 | 0.334±0.143  -0.010/0.612 | -0.074±0.183  -1.133/0.436 | 0.903±0.433  -0.011/1.624 | -0.384±0.235  -0.100/0.727 | -0.049±0.061  -0.099/0.199 |
| HC10 mean±S.D.  Min/Max | 1.208±0.722  0.208/2.384 | 0.472±0.244  -0.024/0.652 | -1.021±0.578  -1.662/0.063 | 0.456±0.212  0.084/0.851 | -1.054±0.515  -1.332/-0.149 | -0.341±0.231  -0.832/0.051 |
| **Total mean±S.D.** | **0.578±0.552** | **0.067±0.319** | **-0.207±0.588** | **0.321±0.563** | **-0.057±0.406** | **-0.194±0.245** |
| Statistics  ASD vs HC | t=0.049, p=0.96 | t=-0.04, p=0.97 | t=-0.07, p=0.94 | t=0.101,  p=0.92 | t=-0.167, p=0.87 | t=-0.139,  p=0.89 |

Independent t-test, ASD: patients with autism spectrum disorder, HC: healthy comparison subjects.

Figure S1. Head motion correction


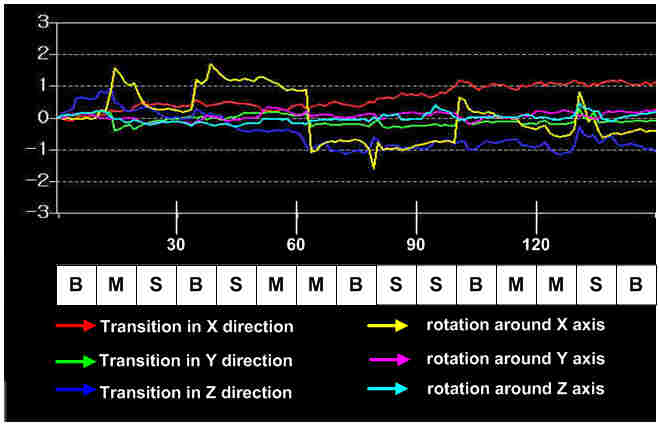


A white cross on a black background (B), a single neutral face (Match, M), and an emoticon stimulation (Stimulation, S)

Figure S2. Comparison of behavioral scores between ASD patients and healthy comparison subjects


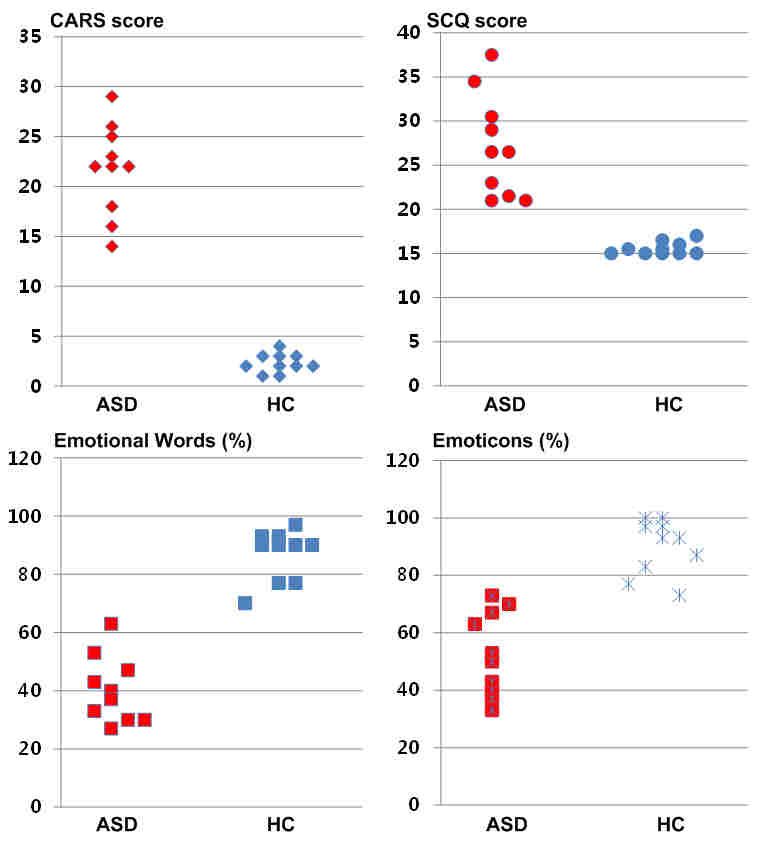


CARS: Child Autism Rating Scale, SCQ: Social Communication Questionnaire

ASD: patients with autism spectrum disorder, HC: healthy comparison subjects

Figure S3. Face emoticons


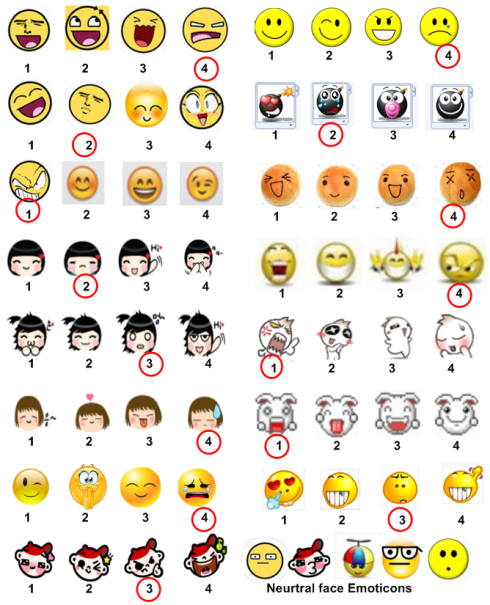


Red circle: unpleasant faces
